# FLORA: A Novel Method to Predict Protein Function from Structure in Diverse Superfamilies

**DOI:** 10.1371/journal.pcbi.1000485

**Published:** 2009-08-28

**Authors:** Oliver C. Redfern, Benoît H. Dessailly, Timothy J. Dallman, Ian Sillitoe, Christine A. Orengo

**Affiliations:** Research Department of Structural and Molecular Biology, University College London, London, United Kingdom; National Cancer Institute, United States of America and Tel Aviv University, Israel

## Abstract

Predicting protein function from structure remains an active area of interest, particularly for the structural genomics initiatives where a substantial number of structures are initially solved with little or no functional characterisation. Although global structure comparison methods can be used to transfer functional annotations, the relationship between fold and function is complex, particularly in functionally diverse superfamilies that have evolved through different secondary structure embellishments to a common structural core. The majority of prediction algorithms employ local templates built on known or predicted functional residues. Here, we present a novel method (FLORA) that automatically generates structural motifs associated with different functional sub-families (FSGs) within functionally diverse domain superfamilies. Templates are created purely on the basis of their specificity for a given FSG, and the method makes no prior prediction of functional sites, nor assumes specific physico-chemical properties of residues. FLORA is able to accurately discriminate between homologous domains with different functions and substantially outperforms (a 2–3 fold increase in coverage at low error rates) popular structure comparison methods and a leading function prediction method. We benchmark FLORA on a large data set of enzyme superfamilies from all three major protein classes (α, β, αβ) and demonstrate the functional relevance of the motifs it identifies. We also provide novel predictions of enzymatic activity for a large number of structures solved by the Protein Structure Initiative. Overall, we show that FLORA is able to effectively detect functionally similar protein domain structures by purely using patterns of structural conservation of all residues.

## Author Summary

Understanding how the three-dimensional (3D) molecular structure of proteins influences their function can provide insights into the workings of biological systems. Structural Genomics Initiatives have been set up to investigate these structures on a large scale and make the data available to the wider biological research community. However, in a significant number of cases, there is little known about the functions of the structures that are solved. To address this, computational methods can be used as a predictive tool to guide future experimental investigations. One such approach is to exploit global structural comparison to assign the protein in question to an evolutionary family, which has already been functionally characterised. However, this is problematic in some large evolutionary families, which contain a number of different functional sub-families. We have developed a new method (FLORA) which is able to calculate 3D “motifs” which are specific to each of these sub-families. Any new protein structure can then be compared against these motifs to make a more accurate prediction of its function. Our paper shows that FLORA substantially outperforms other standard approaches for predicting function from structure. We use our method to make confident functional predictions for a set of proteins solved by the structural genomics projects, which could not have been assigned reliably by global structure comparison.

## Introduction

The prediction of protein function from structure has become of increasing interest as a significant proportion [1] of structures solved by the structural genomics initiatives (SGI) lack functional annotation (for a review see [2]). Furthermore, structure-based approaches are of particular interest for predicting binding sites and/or catalytic sites for the purposes of protein engineering and pharmaceutical development (for reviews see [2],[3]). Many current methods focus on encoding a “template” of functional residues and then aligning this template to whole structures. The problems with taking this approach are deciding what qualifies as a functional residue (e.g. one directly involved in catalysis or ligand binding) and creating biologically-accurate templates for the ever increasing number of available protein structures being deposited in the PDB [4]. Resources such as the Catalytic Site Atlas [5] are carefully curated by hand and restricted to residues directly involved in catalysis, whereas MSDSite [6] and PDBSite [7],[8] generate templates based on active site residues defined in the PDB file by the authors. Although these resources are undoubtedly extremely valuable, it is questionable whether sufficient coverage of the PDB can be maintained when manual intervention is required.

To address the problem of generating templates for all protein structures, there are a number of methods that aim to do this automatically. For example, the reverse template method [1] (available as part of the PROFUNC suite [9]) decomposes a query structure into tri-peptide fragments (putative catalytic triads), which are then matched against a non-redundant set of PDB structures using the search algorithm JESS [1]. Hits are evaluated according to the sequence similarity of the local environment of the template. The GASP method [10] uses a genetic algorithm to construct templates based on their ability to discriminate between different protein families against a background of representatives from the SCOP database [11]. Similarly, DRESPAT [12] implements a graph theoretical approach to discover structural patterns associated with a given family of proteins to locate ligand binding motifs (the PINTS method [13] uses a related approach). MultiProt [14] can provide template of structures through a multiple structure alignment. A recent extension of the Evolutionary Trace method for binding site prediction was used to create structural templates based on predicted functional residues [15]. SiteEngines [16] produces templates by matching the geometry and physico-chemical properties of residues in binding site clefts. As well as atom or residue-level templates, other non-template-based approaches seek to compare the electrostatic properties of binding sites (ef-Site, [17], SURF's UP [18]) or surface accessible clefts which often co-locate with active sites (pvSOAR (CASTp) [19]).

One inherent complexity of using PDB structures to transfer annotations between enzymes is the binding state in which the protein is crystallised — for example, structures crystallised with non-cognate ligands, substrate analogs, transition states or apo-enzymes [20]. As a consequence, precise geometric matching in the active site region can be problematic due to the conformational changes that occur on ligand binding. To address this issue, the methods mentioned above use a variety of approaches such as graph matching or geometric hashing with various tolerance levels. The SOIPPA method [21],[22] takes the alternative approach of using a “geometric potential” to characterise the shape formed by a given set of C_α_ atoms, to account for both local and global relationships between residues across the protein structure. In a recent ligand-binding site comparison analysis, SOIPPA was able to detect distant similarities between very different protein folds binding a range of adenine-containing ligands [21].

Despite the many template methods present in the literature, very few are publicly available to the general user. Hence, the first step in assigning function by structure is often to use global structure comparison methods (e.g. CE [23], DALI [24], CATHEDRAL [25], MAMMOTH [26], FatCat [27], MSDFold [28]), which can detect distant evolutionary relationships even where sequence similarity is weak. These methods have been specifically applied to function prediction (ProKnow [29], Annolite [30]) to assign confidence values when inheriting GO terms between related structures. However, detecting very distant relatives remains a challenge as structure comparison methods generally give an absolute measure (or score) of structural distance, such as RMSD, and applying a cut-off at which one can deduce that two proteins perform related functions results in many missed relationships.

Analyses of CATH [31],[32] have shown that although function and structure are well conserved in the majority of superfamilies, there are a significant number of highly diverse superfamilies where this is not the case [31]. Moreover, the latter superfamilies are disproportionately represented in both the PDB and in the genomes and tend to exhibit a wide range of core biological functions across a large range of species [33]. An analysis by Reeves *et al.* [31] showed that relatives within these superfamilies tend to share a common evolutionary core, but this core is embellished with different insertions of secondary structure elements that often correlate with changes in function. However, although structural embellishments might change some facet of function (e.g. ligand specificity, protein-protein interactions), others have found that relatives can still retain other aspects in common (e.g. catalytic mechanism, such as kinase activity) [34],[35]. Therefore, calculating a global measure of structural similarity or distance (e.g. RMSD) between two proteins can be less informative than focussing on the structural motifs relevant to a given aspect of function.

The FLORA algorithm presented here was designed to derive structural templates for functional sub-groups (FSGs) within diverse CATH superfamilies. FLORA first performs global structure alignment across the superfamily to recognise the distinctive structural patterns associated with each FSG and builds templates based on these patterns. New functional homologues are then detected by using the global structural alignments to relatives in each FSG again, but only scoring the similarity over positions identified by the FLORA motif. This approach performs very well in discriminating between different enzymatic functions, compared to global methods and another motif-based approach. Although we benchmark here on enzyme superfamilies, the method is applicable to superfamilies containing non-enzymatic relatives. To test FLORA, we have automatically generated a large data set of domains from 29 diverse superfamilies (containing multiple FSGs). Our data set allows us to look at the variation of FLORA results between superfamilies and to stress the importance of using a large test data set for benchmarking methods. We have benchmarked FLORA against CE [23], CATHEDRAL [25] and Reverse Templates (RT) [1] to give an indication of how it performs in comparison to other standard methods of function prediction. We also present some examples of structural motifs identified by FLORA and explain their functional relevance. Finally, we use FLORA to make novel predictions of function for proteins solved by the Protein Structure Initiative (PSI).

## Methods

### Generating a data set of functionally diverse domain superfamilies in CATH

**Figure 1:**
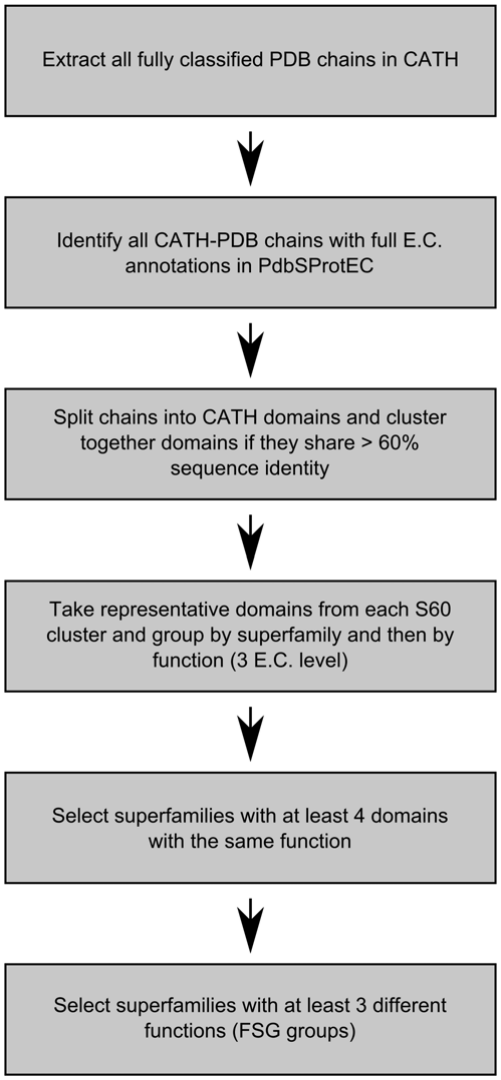
Outline of benchmark data set generation.

In order to benchmark FLORA as a protein function prediction method, it was important to generate a relatively large and unbiased data set. We focussed on functionally diverse superfamilies (≥3 functions at the third E.C. [36] level) in the CATH database, where global fold similarity and evidence of homology is not necessarily indicative of a functional similarity. An overview of the protocol is shown in Figure 1.

All protein chains from PDB structures classified in CATH v3.1 were annotated with an E.C. number using PDBSprotEC [37], which maps PDB chains to corresponding entries in the SwissProt database [38]. E.C. annotations were then transferred from the whole chain level to each constituent domain in a chain. Assigning functional annotation to individual domains is not a straight-forward process, as other domains in the chain (or indeed, residues from other chains in the protein) may be required for the enzyme to be catalytically active. This problem is dealt with more extensively in the PROCOGNATE resource [39]. However, we were only interested in finding domains that were “associated” with proteins of a given enzymatic function, as FLORA was designed to consider all residues for inclusion in a template and not just those in the active site.

To simplify the benchmark data set, all domains from enzymes assigned more than one E.C. (i.e. multifunctional enzymes) were removed. This exclusion criterion removed less than 8% of enzymatic chains in the PDB. In addition, any domains with an incomplete E.C. number (e.g. 2.7.-.-) were also excluded.

All annotated domains in CATH were clustered at 60% sequence identity and a representative taken from each cluster (S60Rep). This threshold was applied as 60% has been found to be an appropriate sequence cut-off for functional similarity [40],[41]. Discovering homologous domains sharing more than 60% sequence identity is trivial using BLAST [42] and other sequence-base methods and we wished to generate a benchmark data set that contained more challenging cases.

S60Reps were then grouped within the superfamily if they shared at least the first three E.C. numbers; to create what we will subsequently refer to as a functional sub-group (FSG). A CATH superfamily was then included in the data set if it contained at least 3 FSGs, where each enzyme family contained at least 4 S60Reps. These criteria were chosen to create a sufficiently diverse data set, which could be effectively assessed using leave-one-out benchmarking.

The final domain data set (Dataset S1) comprised: 82 FSGs from 29 different CATH superfamilies (a total of 911 S60Reps domains), covering all 3 major protein classes (α, beta and mixed α-beta). Although the data set comprises ∼2% of the total number of superfamilies in CATH, these superfamilies account for ∼48% of domain sequences from functionally diverse superfamilies in Uniprot. Furthermore, they represent a set of domains where global fold similarity does not necessarily correlate with functional similarity.

### The FLORAMake algorithm

**Figure 2:**
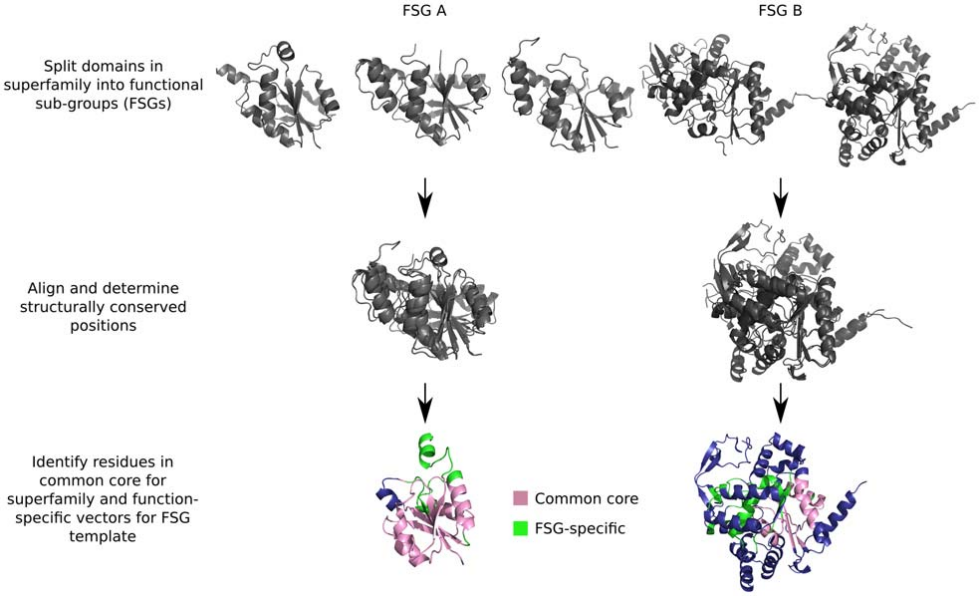
Graphical outline of FLORAMake algorithm.

An outline of the FLORAMake algorithm is shown in Figure 2. The aim was to select a set of conserved vectors from a given domain in a given FSG which when compared against relatives of different functions/FSGs would produce a low score and similarly a high score to relatives with the same function.

#### Align all domains in the superfamily using CATHEDRAL

Methods which attempt to create structural templates of residues associated with a given function rely on a range of methods [1],[12],[15],[16] for focussing on functionally relevant regions of the protein. These targeted methods can therefore be used to detect common motifs when calculating global structural similarity might fail [25], but the performance is partially dependent on the accuracy at which they predict functional residues. The aim of FLORA was to explore the whole protein domain to detect structural regions important for the common functional roles of domains in the FSG. To do this FLORA does not focus on predefined sites but performs global structure comparisons across a given superfamily to attempt to identify “hotspots” which are specific to a particular FSG. To perform the global structure comparisons, FLORA exploits the CATHEDRAL method (see Text S1). Compared to other structure comparison methods, CATHEDRAL has been shown to align the largest proportion of equivalent residues with respect to manually curated alignments [25]. Therefore, by using CATHEDRAL to align relatives, FLORA would be able to consider a larger number of positions that could be functionally-relevant for a given FSG.

The first step in our protocol was therefore to generate structural alignments using CATHEDRAL between *all* pairs of domains within each superfamily in the data set.

#### Identify structurally conserved residues

All pairs of structure-structure alignments between domains in a given FSG were analysed to identify aligned residues. A set of residues for each domain was then generated from the pairwise alignments to include only those residues that were aligned to residues in at least 75% of other domains in the FSG (to account for sub-optimal alignments). A cut-off of 75% was chosen after exploring a range of cut-offs (0–100%) and gave the fastest performance without affecting the precision/recall of FLORA. These were designated rescons positions.

#### Calculate vectors between conserved residues

For each domain, vectors were calculated between all rescons positions. To allow vectors to be appropriately compared between domains, a vector was calculated between the C_β_ atoms of residues A and B and then multiplied by a co-ordinate frame calculated from the tetrahedral geometry of the bonds of the C_α_ of residue A as described in [43]. As the C_α_ geometry of residues A and B are not identical, vectors were calculated in both the A→B and B→A direction. However, we found that taking only one of these vectors forward to the next steps in the algorithm gave the same performance as using both, but increased the speed of FLORA by halving the number of vectors that needed to be analysed.

#### Compare vectors across the superfamily

A given vector from a domain in the FSG was compared to equivalent vectors in domains across the whole superfamily. Equivalent vectors were obtained from the CATHEDRAL structural alignment of the domains being compared. For example, residues 93 and 105 in CATH domain 1vl2A01 are equivalent to residues 92 and 108 in 1k92A01 according to the structural alignment. Hence, the vectors 93→105 (v1) and 92→108 (v2) were scored for similarity using the formula below (which is identical to the vector score developed for the SSAP [43] and CATHEDRAL [25] algorithms). We experimented with different values of a and b, and found that a = b = 2 gave the best performance (interestingly these were the values used in the original implementation of SSAP [43]).
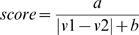



#### Determine vectors that are more conserved within a given functional sub-group (FSG)

The next step in the algorithm is to determine vectors for a given domain which are more similar to equivalent vectors in other domains in the same FSG than to those of relatives in the superfamily with different functions (i.e. in different FSGs). The aim was to eliminate vectors that are conserved mainly to preserve the common fold of the superfamily. Two distributions were calculated for each vector: a) scores to domains in the same FSG (DIST-F) and b) scores of domains in different FSGs (DIST-S). The means of DIST-F and DIST-S were then calculated and the vector was initially determined to be FSG-specific if it satisfied the following inequality:
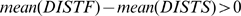
(1)


We experimented with various statistical tests (e.g. Wilcoxon rank sum, calculating an empirical p-value), but found that the set of selected vectors could be best reduced by jack-knifing the data set and repeating the calculation above. That is, each domain in the training set was removed in turn and FLORA only selects a vector if the inequality is always satisfied.

We also explored incorporating measures of sequence similarity when scoring vectors, but in our hands this degraded the performance of FLORA. This could be due to the fact that the benchmark data set contained very diverse relatives and hence exploring the sequence signal requires a more sophisticated approach.

#### Store function-specific vectors for each domain

At this point, each domain in the FSG is associated with a set of FSG-specific vectors, which we termed the “FSG-domain template set”.

### Scoring domains against templates (FLORAScan)

#### Matching and scoring FLORA templates

To score a given query domain against the template for a given domain in a given FSG relies again on the global structural alignment by CATHEDRAL. Hence, the first step is to align the query domain against an FSG domain but then only score the similarity across the subset of template vectors. Essentially, we are calculating a local score over the FLORA template from the correspondence determined by a global structural alignment. Each vector in the template set associated with the FSG domain is scored against the equivalent vector in the query domain (using equation 1), based on the aligned residues from the global alignment. Any vectors that are not aligned (i.e. gapped positions) are given a score of zero. The total similarity of the query domain against enzyme domain (the florascore) is simply the sum of these similarities, normalised by the total number of vectors in the template (Equation 2).
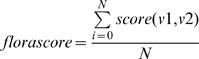
(2)Where N = number of template vectors; v1 = template vector; v2 = equivalent vector in query domain

#### Conversion to Z-score

We hypothesised that the extent to which the structure of a domain can change before its enzymatic function changes might be specific to the homologous superfamily. For each FLORA domain-function template, a distribution of all scores is calculated against all domains in different FSGs. The florascore between a given pair of query and enzyme domains is then transformed into a Z-score.

### Leave-one-out benchmarking

As FLORA is essentially a pattern discovery method, it was vital to assess its performance in an unbiased fashion. We took a standard leave-one-out (or jack-knifing) approach as is often used to test machine learning methods. For each superfamily, one test domain was removed, while training on the remaining domains. The test domain was then scored against all the resulting templates. The aim of this process to was accurately reproduce a situation where a novel domain is classified into a CATH superfamily and then needs to be assigned to a functional group.

### Analysis of the performance of function prediction methods

The performance of FLORA, CATHEDRAL [25], CE [23] and Reverse Template (RT) [1] were analysed by plotting sensitivity (i.e. tp/(tp+fn)) versus precision (tp/(tp+fp)). We compared the performance on individual superfamilies by calculating AUC value (area under ROC curve).

### Comparison of FLORA motifs to known functional residues

In order to examine where residues identified by FLORA overlapped with known functional residues, we compared the location of FLORA positions to those in the Catalytic Site Atlas [5] (v2.2.9).

For each functional sub-group (FSG), we selected the domain that had the highest mean global structural similarity (measured by CATHEDRAL) to all other members of the FSG as a representative. All residues, from each relative within an FSG, identified by FLORA and CSA annotations were then mapped onto this representative using the CATHEDRAL structural alignment. Consequently, for each FSG we had a representative structure where all residues were annotated as FLORA positions, catalytic residues, or neither. The CSA provided annotations for 61 out of 82 FSGs (74%). We then calculated the average distance between the FLORA residues to the catalytic residues and the average distance between non-FLORA and the catalytic residues.

### Analysing function-specific regions identified by FLORA

FLORA produces a set of inter-residue vectors for each domain in a given FSG that are considered to be specific to its enzymatic function, in the context of its evolutionary superfamily. In order to visualise where these vectors lay, we took each set of domain templates for a given enzyme family and mapped them onto the most representative structure — i.e. the structure with the greatest cumulative global structural similarity to all other domains in the family. A given residue was then coloured if it was involved in the top 30% of FLORA template vectors. Residues that are conserved across the whole superfamily (in 75% of relatives) were also identified and those which overlapped with FLORA residues were coloured gold.

### Function prediction for PSI structural genomics targets

Despite targeting proteins with no significant sequence similarity to existing structures in the PDB, Protein Structure Initiative (PSI) structures can often be classified into one of the large, diverse superfamilies in CATH by structure comparison methods once their structure has been solved. However, these superfamilies contain a significant number of relatives with different functions and therefore to be able to further assign these proteins to a specific functional sub-group is of great use for guiding future functional studies. We took all PSI structures solved up to January 2008 that had been newly classified in v3.2 of the CATH database and selected the 276 domains which fell into one the superfamilies in our data set. These 276 were further clustered at 60% sequence identity to produce a non-redundant test set of 104 domains, which was then scanned against the FLORA templates for each FSG in order to predict their function. To exclude hits that could have been fairly confidently assigned using global structure comparison, we removed any structures that matched a CATH domain in v3.1 library with a SIMAX score<1.5 [25].
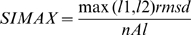



## Results

FLORA was designed as a generic method to create structural motifs that can discriminate between different functional sub-groups (FSGs) within diverse domain superfamilies, purely using patterns of structural conservation — FLORA makes no assumptions as to the physico-chemical properties of functionally important residues and uses a purely structure-based conservation score (i.e. sequence similarity is not used to select or score equivalent motif vectors, see Methods). We created a benchmark data set of diverse enzyme superfamilies in the CATH database [44], although FLORA can be applied to protein structures grouped by any function or superfamily annotation scheme.

We tested the performance of FLORA against global structure comparison methods (CE [23], CATHEDRAL [25]) and the Reverse Template (RT) method [1]. The residue positions identified by the FLORA templates were examined to determine whether they co-located to functional regions in the protein structures. Finally, we used FLORA to predict broad enzymatic functions for a set of structural genomics targets solved by the Protein Structure Initiative [45].

### How well does global structural similarity (CATHEDRAL and CE) predict membership of functional sub-groups (FSGs)?

To fairly benchmark any function prediction algorithm, it is important to compare against current methods. Unfortunately, the vast majority of function prediction methods are not publicly available, however here we compare against CE as this method has been used as a benchmark for other structure-based function prediction methods (e.g. [10],[21]). We also compare the performance of FLORA against a more sensitive structure comparison method (CATHEDRAL [25]) and a leading function prediction method (RT [1]).

**Figure 3:**
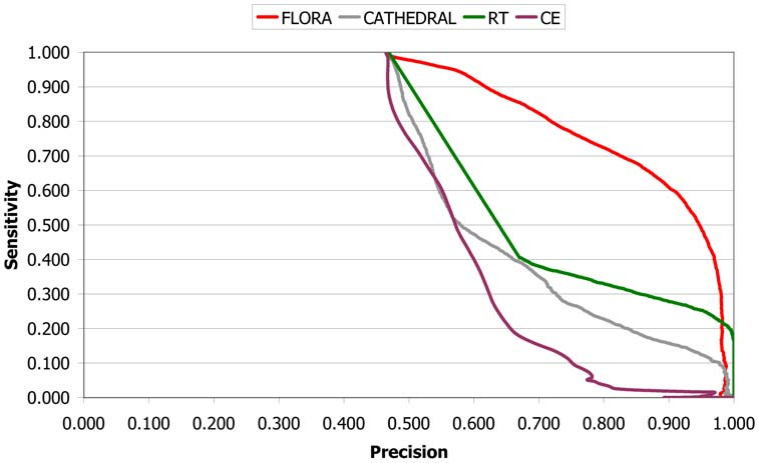
Graph of sensitivity versus precision to show the performance of CE, CATHEDRAL, RT and FLORA for the prediction of enzyme family.

Initially, we investigated to what extent global structure comparison could be used to reliably assign function. The graph of sensitivity versus precision (Figure 3) shows the ability of CE and CATHEDRAL to discriminate between domains in the same enzyme family across our entire data set. It can be seen that at high precision (∼90%), CATHEDRAL outperforms CE, although the sensitivity is still very low (18%). We suspect that the superior performance of CATHEDRAL over CE is due to the fact that it is able to generate improved alignments of homologous structures by aligning more equivalent residues (as shown in [25]). The performance of both methods shown here is fairly poor for correctly classifying domains into FSGs, but it is obviously important to note that neither of the methods was designed to detect functional relationships.

### Using FLORA to predict membership of functional sub-groups

FLORAMake and FLORAScan were applied to the domain data set and the performance was assessed using a leave-one-out approach (described in the Methods section). It can be seen from Figure 3 that even at high precision, FLORA significantly outperforms CATHEDRAL, CE and RT — e.g. 90% precision, CATHEDRAL detects only 15% of true functional homologues, versus 27% for RT and 61% for FLORA. These results show that the FLORA algorithm significantly outperforms global structure comparison. This can be explained by the fact that although FLORA uses the same alignments as CATHEDRAL, it only scores those positions which have been identified as functionally-relevant (i.e. captured by the FLORA template) within a given FSG. Furthermore, FLORA uses data from multiple structures and is able to accurately discover functionally-relevant structural motifs and discover more than twice the number of functional homologues at 90% precision than RT. This suggests that where the data are available, exploiting multiple structures with similar functions can improve the sensitivity of function prediction methods. However, where these is not available, methods such as RT [1] can be very valuable.

### How does the performance of FLORA vary between superfamilies?

**Figure 4:**
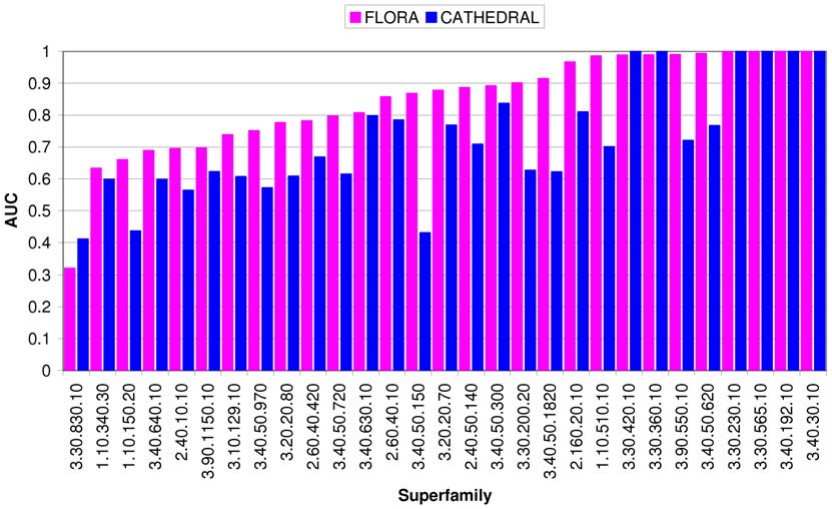
Histogram of the performance of FLORA versus CATHEDRAL for individual superfamilies, assessed using the AUC (area under ROC curve) statistic. The superfamilies were ranked according the AUC, with the worst performing listed first.

**Figure 5:**
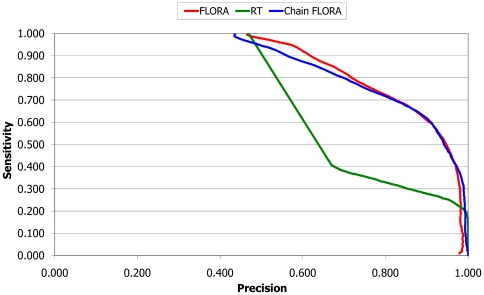
Graph of sensitivity versus precision to show the performance of using FLORA at the domain level and chain level. The performance of RT (which works at the whole chain level) is shown for comparison.

FLORA was benchmarked on 29 functionally diverse enzyme superfamilies and the performance quoted thus far refers to an average calculated over the entire data set. Figure 4 shows the performance per superfamily (as measured by the Area Under ROC Curve (AUC)) for FLORA and CATHEDRAL. It can be seen that where FLORA is able to perfectly discriminate between domains in different functional sub-groups (i.e. AUC = 1.0), CATHEDRAL is also able to do so as functionally-similar domains must share high global structural similarity. However, for all but one (CATH code: 3.30.830.10) of the superfamilies in the data set, FLORA out-performs CATHEDRAL. Superfamily 3.30.830.10 comprises two FSGs (aminopeptidases and carboxypeptidases), which contain domains that are part of larger multi-domain complexes. For example, the protein chain 1hr6A actually contains two homologous yet non-identical domains (<30 sequence identity), both of which are members of this superfamily — i.e. a domain duplication has produced the multi-domain architecture 3.30.830.10::3.30.830.10. As a consequence, it is more biologically meaningful to align this superfamily at the chain level, which indeed improves the performance of FLORA (AUC increases from 0.32 to 0.88, see next section and Figure 5). Although there is only one example of this case in our data set, it will be important to account for domain duplications when building templates in the future. For example, we encountered similar problems in a superfamily of periplasmic binding domains (CATH 3.40.190.10), where a domain duplication creates a receptor of two halves involved in the transportation of small ligands (unpublished data).

### Comparison of the performance of FLORA using single domains versus whole protein chains

At this point, it can be seen that simply focussing at the domain level FLORA is able to very effectively improve the recognition of structures in the same FSG. This is interesting given that the majority of structure-based function prediction methods tend to use the whole protein chain. A possible explanation of the power of FLORA could be that the domains in our data set form a core part of the enzymatically active region of the whole protein. Alternatively, it could be that the selected vectors for each template also contain residues that interact with other enzymatic domains within the chain, and it is these interaction sites that FLORA is detecting.

To see whether any improvement could be achieved by using the whole protein chain, we used CATHEDRAL to re-align the corresponding PDB chain for each of the domains in the data set and performed an identical benchmark as before. Figure 5 shows that the performance increase of using whole chains over using the component domains is minimal. This suggests that there is enough of a structural signal at the domain level and adding vectors from other domains in the protein chain does not seem to be advantageous. It also means that FLORA could be used to transfer functional annotation between relatives with different multi-domain architectures, therefore expanding the scope of the method.

### Where do FLORA template residues lie on the structure?

The benchmarking analysis presented above shows that FLORA is indeed able to correctly discriminate between homologous domains from different FSGs better than global structure comparison, despite using global alignments to determine residue correspondence. This suggests that although a global alignment may not be perfect, especially between very distant relatives, it still aligns enough residues that are important for maintaining different functions. To examine where these function-specific residue lay, we chose a representative structure for each enzyme family and visualised the conserved FLORA residues (see Methods section).

We have analysed these motifs further in domains from the HUP superfamily (CATH 3.40.50.620 [46]), which is the subject of particular attention within our group. HUP domains are very diverse in terms of sequence, structure and function, and are involved in various essential biological processes (e.g. protein translation). In addition, several proteins with HUP domains have attracted attention due to their medical importance (e.g. [47]). Domains in this superfamily adopt a Rossmann-like fold with a central parallel β-sheet surrounded on both sides by α-helices. The main active site is always located in the C-terminal half of the central β-sheet and is generally involved in nucleotide-binding. HUP domains in the FLORA dataset divide into 3 major FSGs when clustered using the first three digits of the E.C. numbers. In the following section, we consider one representative member of each of these FSGs to describe motifs identified by FLORA.

**Figure 6:**
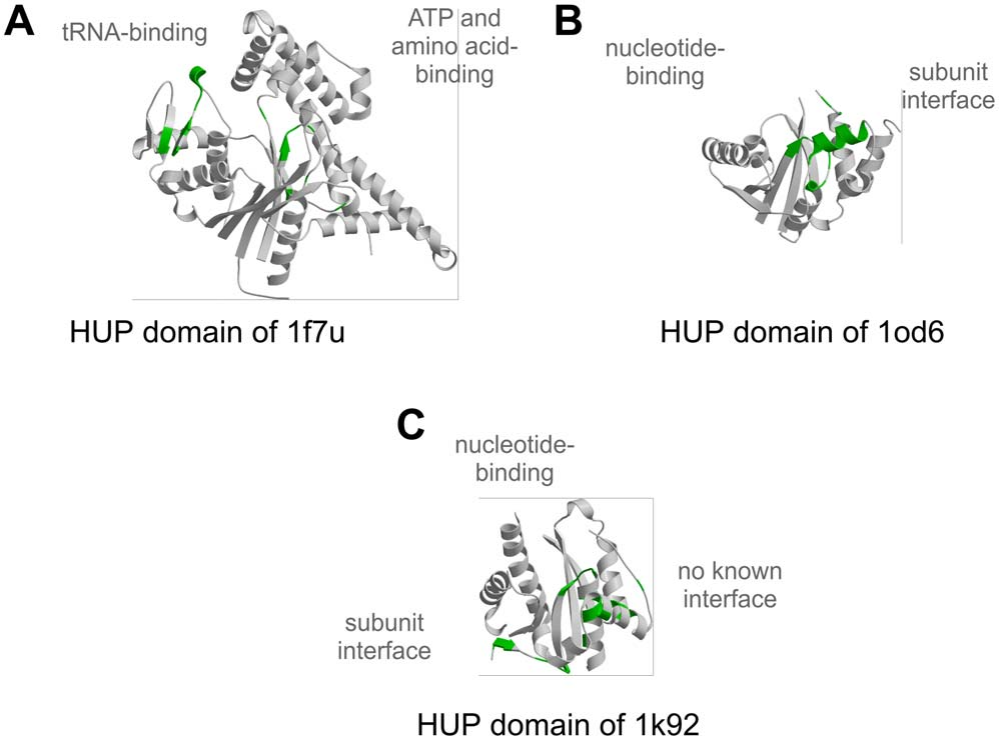
Representatives from the Tyrosyl-Transfer RNA Synthetase superfamily (3.40.50.620) in CATH. A 1f7u, B 1od6, C 1k92. FLORA residues are shown in green.

The first FSG consists of the catalytic domain of class I aminoacyl-tRNA synthetases (EC 6.1.1.-). These enzymes are essential for protein translation as they catalyse the ligation of amino-acids to their cognate tRNAs in a two-step mechanism that involves ATP. The HUP domains of aminoacyl-tRNA synthetases are found in many different multi-domain contexts in CATH, which appear to partially depend on the amino-acid substrate (data not shown). In representatives from this group, (*S. cerevisiae* arginyl-tRNA synthetase, PDB: 1f7u), FLORA identifies two major motifs, one of which is located in the amino-acid and ATP binding site, whereas the other covers residues in loops that bind the tRNA (Figure 6A).

The next FSG in the HUP superfamily is a group of metabolic enzymes called nucleotidyltransferases (EC 2.7.7.-), which transfer nucleotidyl groups from nucleotide tri-phosphates to other compounds. The nucleotidyltransferase we have analysed further (*Th. Thermophilus* pantetheine phosphate adenylyltransferase PDB: 1od6), is a relatively small protein and consists of a homo-hexamer of single HUP domain subunits. FLORA identifies two motifs in this domain, one of which locates in the main active site in the C-terminal half of the central β-sheet, whereas the other maps to the inter-subunit interface (Figure 6B).

Finally, the third FSG consists exclusively of argininosuccinate synthases (EC 6.3.4.5), which catalyse the ATP-dependent synthesis of argininosuccinate from citrulline and aspartate. These enzymes are homo-tetramers in which each subunit is comprised of a nucleotide-binding HUP domain and an additional domain involved in multimerisation and catalysis. Three motifs are identified by FLORA in *E. coli* argininosuccinate synthase: one is located in the nucleotide-binding site (C-terminal half of the central β-sheet), another consists of residues at the interface with other subunits of the tetramer, whereas the third motif is comprised of residues from N-terminal α-helices that are not involved in any identified interactions to our knowledge (Figure 6C). The location of these α-helices on the outward surface of the tetramer cannot exclude the possibility that these FLORA residues might be involved in interactions that have yet to be described in the literature.

Analyses of residues identified by FLORA in these domains and others in this superfamily (data not shown) suggest that FLORA is generally able to target motifs known to be involved in different aspects of molecular function, like binding interfaces or catalytic sites. This behaviour is somewhat expected from FLORA, which was specifically designed to detect such function-related signatures in homologous domains. By mapping catalytic residues from the CSA onto each FSG representative (see Methods), we found that in 59% of cases the FLORA residues were closer to the functional site than other residues in the domain. This is interesting as it means that in a significant number of FSGs, FLORA is identifying other positions in the protein, for example those involved in interaction sites as demonstrated by the examples discussed above. In the particular case of the HUP superfamily mentioned above, it is noteworthy that in each FSG, FLORA not only identifies functional regions which are unique to the FSG (e.g. the tRNA binding site in aminoacyl-tRNA synthetases), but also residues in the main nucleotide-binding active site which is shared by HUP domains from all FSGs at the C-terminal half of the central β-sheet. Although this would require further investigation, it suggests that FLORA is able to detect relatively small differences in residue positions and orientations between similar active sites in different FSGs.

Examining similar representatives from the Class I aldolase superfamily (3.20.20.70) reveals that FLORA template residues (Text S1) tend to cluster around the active site of the enzymes (data on active site residues from the Catalytic Site Atlas [5]), which suggest that it is where the majority of structural features characteristic of each FSG occur.

### Using FLORA to predict functions for PSI structures

Our analysis thus far has shown that FLORA is able to substantially improve on the performance of global structure comparison for reliably assigning domains to functional sub-groups. We therefore sought to use it to make novel predictions for structural genomics targets from the PSI. As a data set, we took structures that had been assigned to superfamilies in the latest version of CATH (v3.2) and scanned these against the FLORA templates. Using the benchmark curve from the leave-one-out benchmark, we took a score cut-off corresponding to a precision of 95% (Z-score>3.4) to ensure high confidence in our assignments. All hits above this cut-off were collated, rather than simply taking the top hit so that we could account for bi-functional enzymes and observe any conflicting predictions (i.e. those structures which hit more than one FSG template). A complete table of results is shown in Text S1.

104 domains from our v3.2 PSI set correspond to 94 PDB structures. Of these 94, we were able to make predictions for 66 (70.4%) with FLORA. To assess the added value of using FLORA over global structure comparison, we took out any PSI structures that matched a domain in CATH with a SIMAX score<1.5 (see Methods). This left us with 51/66 (78%) predictions that could not be easily assigned with CATHEDRAL. This supports the earlier benchmark of FLORA, which shows that scoring structural similarity over all FSG-specific residues can dramatically increase the number of functional homologues we are able to detect.

**Figure 7:**
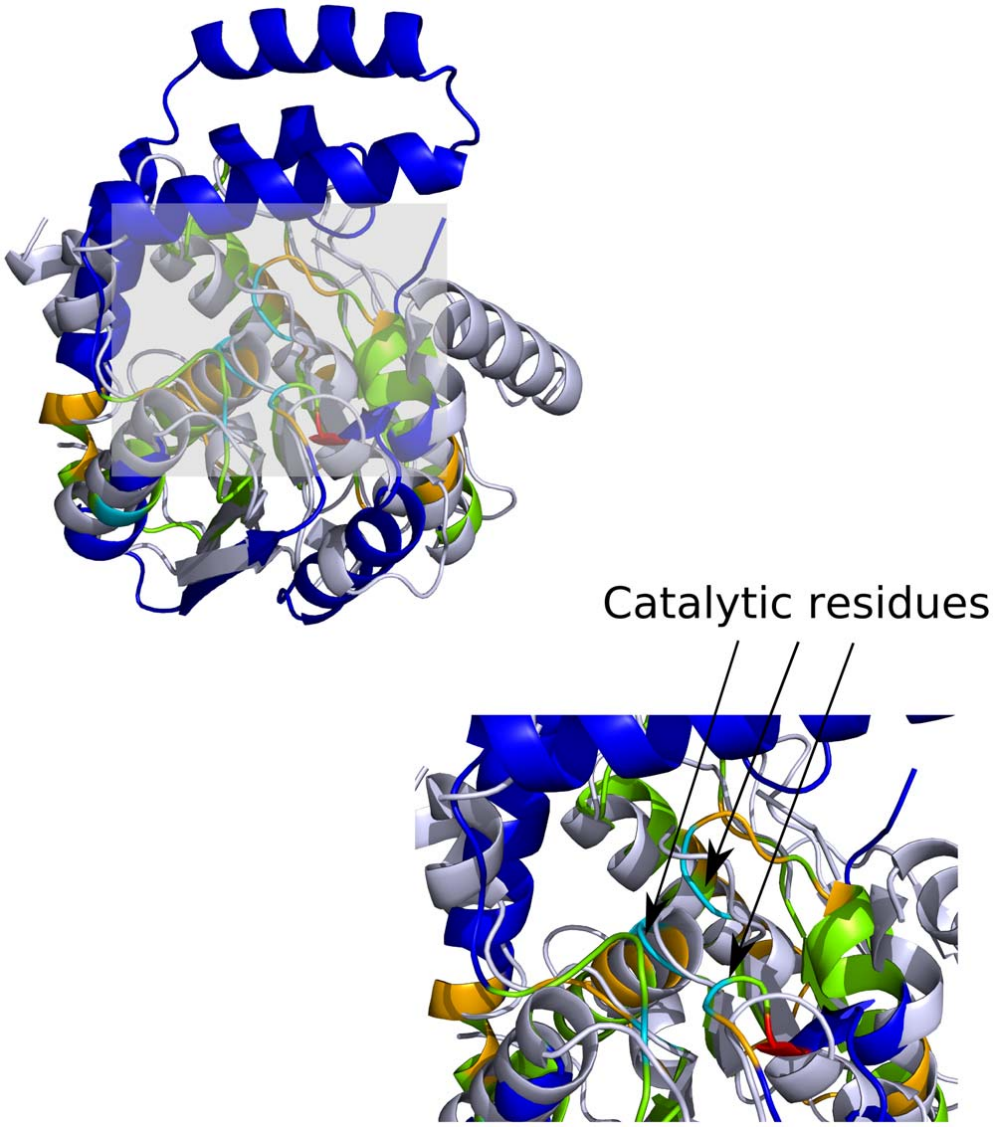
Superposition of PSI structure 2pbl (dark grey) with 1tqh (superfamily 3.40.50.1820, EC 3.1.1.-). FLORA positions are coloured as in previous figures and catalytic residues are shown in light blue. It can be seen that there is reasonable agreement in the region of the active site.

Figure 7 shows the structure of 2pbl (a putative thiol esterase from the Joint Center For Structural Genomics) superposed against its best hit 1epx. A closer superposition of the active site shows conservation of the surrounding secondary structures and even the positions of the catalytic residues. FLORA finds significant hits to all members of the FSG (E.C. 3.1.1-, Carboxylic ester hydrolases) in superfamily 3.40.50.1820, despite none of the domains superposing with an RMSD less than 4, indicating that 2pbl is a distant relative of other superfamily members. The other FSG in the superfamily corresponds to E.C. 3.4.16.-, which is a group of Serine-type carboxypeptidases to which FLORA assigns no significant hits. FLORA predicts 2pbl to be a carboxylic ester hydrolase, as opposed to a Thiolester hydrolase (E.C. 3.1.2) as suggested by the authors. However, given that there are no examples of thiolesterases currently in the superfamily it is possible that they are in fact closely related to the carboxylic ester hydrolases. Biochemically, this function is certainly closer than the peptidase function of FSG (EC 3.4.16.-).

FLORA predicted NESG structure 2bdt with the E.C. number 2.7.1.-, which is a group including enzymes such as fructose 1-,6 bisphosphate. When this structure was published, it was assigned as a putative gluconate kinase but currently has no official E.C. annotation.

PDB 1vm8 from the JESG consortium was functionally characterised when the structure was solved as UDP-n-acetylglucosamine pyrophosphatase and given the E.C. number E.C. 2.7.7.23. Again, FLORA correctly predicts the E.C. number as 2.7.7.-, despite low global structural similarity to any domains in the template data set.

1ylo is a hypothetical protein solved by the MCSG consortium in 2005. FLORA predicted the E.C. number 3.4.11.-, which comprises a group of amino-acid specific peptidases, with significant hits (Z-score>4) to three domain templates in our data set. A BLAST search indeed reveals significant hits (>99% sequence identity) to annotated amino peptidases, as the protein has now been functionally characterised since its structure was solved. Again, these trivial hits were not in the data set we used, which demonstrates the power of FLORA to find functional homologues even after significant evolutionary divergence.

## Discussion

FLORA is a novel algorithm which exploits patterns of structural conservation to derive templates for different functional sub-groups (FSGs) within diverse domain superfamilies. Unlike many other methods which focus on generating templates based on known or predicted functional residues [1],[10],[15], FLORA considers all residues to provide a more discriminating functional fingerprint. We have shown it is able to use these templates effectively to discriminate between domains with different functions better than global structure comparison (CATHEDRAL), CE and RT.

By generating a superfamily-specific Z-score, we found that the performance of FLORA increases significantly. This suggests that the degree of structural variation that confers a change in function is specific to each superfamily and the absolute structural similarity must be compared to a background distribution. Therefore, as has also been identified at the sequence level [40],[41], function prediction methods should account for the divergence of the superfamily, rather than adopt one similarity measure that applies to all superfamilies. However, we acknowledge that a representative distribution can only be obtained in sufficiently populated superfamilies.

Another important novelty in our approach was to create a large data set comprising 29 superfamilies (which is made publically available). Although FLORA performed well across the majority of superfamilies, this was not universally true, which suggests that function prediction methods should be benchmarked across as diverse a data set as possible. We have also shown that CATHEDRAL outperforms CE, probably due to producing superior alignments outside of the conserved structural core. Although global structure comparison is not always able to reliably find distant functional relatives, we feel it is appropriate for benchmarking new methods to give a guide of the value they add to structure-based function prediction.

As detailed in the methods, FLORA calculates vectors based on the geometry of C_β_ side chain atoms. However, a re-implementation using just C_α_ co-ordinates produces almost identical performance on the data set (data not shown). This is encouraging as it increases applicability of our method to theoretical and homology-based models.

One of the major ways in which FLORA differs to other methods is by focussing on the domain, rather than at the whole chain or protein complex level. Simply because a domain is present in a given enzyme does not necessarily mean it contributes to or confers catalytic activity. Indeed it might be responsible for protein-protein interactions or other aspects of function, such as locating the protein in a given part of the cell. We have shown that except in the case where there has been a domain duplication (superfamily 3.30.830.10), deriving structural motifs at the domain level performs as well as aligning whole multi-domain chains. Our hypothesis is that where FLORA does not locate conserved positions around the active site, it is able to find parts of the domain that interact with other catalytic domains. We intend to undertake more detailed analysis of other CATH superfamilies to confirm this.

FLORA makes no assumptions about the physico-chemical (e.g. solvent accessibility or polarity) or sequence conservation properties of residues in the templates it derives, only that they show high structural conservation within a given functional sub-group. As a consequence, we observed residues both around the enzymatic active sites and in other locations in the protein. In two of the example superfamilies presented here, we have shown that FLORA template vectors co-locate around the active site. This is possibly due to structural changes in the protein that allow for different relatives to bind different ligands. However, this trend is not observed across the whole data set, where only 59% of FLORA template vectors are on average closer to the active site than other residues in the protein. This suggests that it is not only the enzymatic site that is important for discriminating between different FSGs, but other locations in the structure related to domain-domain or protein-protein interfaces.

The substantial improvement in performance of FLORA over global structure comparison has allowed us to assign 70% of structural genomics targets, assigned to superfamilies in our data set to functional sub-groups, in this case predicting the type of catalytic reaction they perform. Of our FLORA predictions, 78% could not have been reliably made by standard structure comparison techniques, as we were able to transfer annotation from far more distant relatives (RMSD>4 Å). Although some of the predictions we made are supported by experimental work that occurred after the structure was solved, the accuracy of the rest remains for future functional characterisation work.

Taken in the context of our previous analysis of functional divergence across large domain superfamilies in the CATH database [31], we have shown that it is indeed possible to derive structural templates that can be used to characterise these different functional sub-groups, without explicitly focussing on known or predicted catalytic residues. Both CATHEDRAL and FLORA exploit the same algorithm to align structures, but the performance increase observed by FLORA is due to the fact that it identifies those positions which are distinctive to a function group and only scores the structural similarity over these positions, whereas CATHEDRAL calculates a global score. Although we have benchmarked here using CATH enzyme superfamilies, FLORA can be applied to any other functional or superfamily classification (both enzyme and non-enzyme) where there are sufficient structural data. We are currently implementing FLORA as a web service for the structural biology community.

## Supporting Information

Dataset S1 Benchmark data set for FLORA.
(0.08 MB XLS)

Text S1 Supporting Information.
(0.31 MB DOC)
