# Supplementary material for: FLORA: A Novel Method to Predict Protein Function from Structure in Diverse Superfamilies
Source: PLoS Comput Biol. 2009 Aug 28;5(8):e1000485. doi: 10.1371/journal.pcbi.1000485 (PMC2721411; doi:10.1371/journal.pcbi.1000485)
Supplement: Text S1 — Supporting Information. (0.31 MB DOC) [file pcbi.1000485.s002.doc]

**Description of CATHEDRAL algorithm**

CATHEDRAL is a structure comparison algorithm that uses a combination of a fast secondary structure comparison approach and a slower, but more accurate, residue-based double dynamic programming.

The query structure is first aligned using the basis of its secondary structures (GRATH, Harrison et al. 2003). Matches are then identified if they have a significant E-value to the query. The aligned secondary structures are then used to guide a residue-based alignment using a modified version of the SSAP double dynamic programming (DDP) algorithm (Orengo and Taylor, 1989). The DDP step takes pairs of potentially equivalent residues, calculates vectors between their C-beta atoms and scores the similarities between these vectors. All scores are then used to fill a matrix of similarities and calculate an alignment. This procedure is performed for all residue pairs with similar secondary structure and solvent accessibilities. All high scoring alignments are then accumulated in a summary matrix, which is again solved by dynamic programming to produce a final alignment. For further details, see Redfern *et al.* 2007 and Orengo and Taylor, 1989.

| 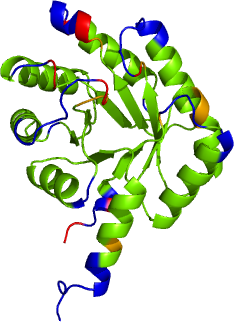  Ligand binds | 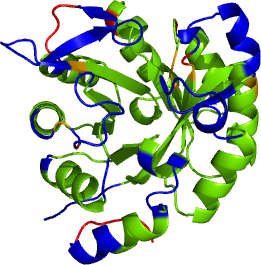  Ligand binds |
| --- | --- |
| **1mzhA0** | **1h5yB00** |
| 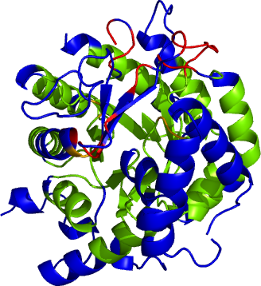  Ligand binds |  |
| **1d3gA01** |  |

Supplementary Figure 1 Representatives from the Class I aldolase superfamily (3.20.20.70) in CATH. FLORA residues are shown in red, common core residues are shown in green and those residues that fall into both categories are shown in gold.

| **PSI target PDB** | **Predictions (query domain:hit domain:FSG:FLORA Z-score)** |
| --- | --- |
| 1npd | 1npdB01:1vi2A01:3.40.192.10_1.1.1:281.947 |
| 1tlt | 1tltA01:1cf2O01:3.40.50.720_1.2.1:4.48053 |
| 1vl0 | 1vl0A01:1kbzA01:3.40.50.720_1.1.1:5.92895 |
| 1vl2 | 1vl2C01:1vl2A01:3.40.50.620_6.3.4:55.464 |
| 1vm8 | 1vm8B01:1jv1A01:3.90.550.10_2.7.7:91.9597 |
| 1ylo | 1yloA01:1cg2A01:3.40.630.10_3.4.17:5.01417 |
| 1z9f | 1z9fA00:1eovA01:2.40.50.140_6.1.1:7.08011 |
| 2ess | 2essA01:1s5uE00:3.10.129.10_3.1.2:63.0451 |
| 2f6r | 2f6rA00:1uf9A00:3.40.50.300_2.7.1:6.62724 |
| 2fa8 | 2fa8B00:1n8jA00:3.40.30.10_1.11.1:5.71969 |
| 2fiq | 2fiqB01:1dosA00:3.20.20.70_4.1.2:5.21393 |
| 2glf | 2glfA01:1y7eA01:3.40.630.10_3.4.11:10.7161 |
| 2gre | 2greA01:2gljA01:3.40.630.10_3.4.11:4.32387 |
| 2grj | 2grjA00:1jjvA00:3.40.50.300_2.7.1:3.95097 |
| 2hbo | 2hboA01:1s5uE00:3.10.129.10_3.1.2:20.8518 |
| 2hc9 | 2hc9A02:1gytL02:3.40.630.10_3.4.11:12.4295 |
| 2hdw | 2hdwA01:1zi8A00:3.40.50.1820_3.1.1:9.45494 |
| 2hjs | 2hjsA02:1b7gO02:3.30.360.10_1.2.1:104.403 |
| 2hlj | 2hljA01:1s5uE00:3.10.129.10_3.1.2:55.0416 |
| 2hma | 2hmaA01:1k92A01:3.40.50.620_6.3.4:5.42946 |
| 2ho3 | 2ho3B02:1lc0A02:3.30.360.10_1.3.1:5.29306 |
| 2hx5 | 2hx5A00:1s5uE00:3.10.129.10_3.1.2:67.171 |
| 2i3d | 2i3dB00:1cex000:3.40.50.1820_3.1.1:11.0552 |
| 2i5e | 2i5eA01:1eziA00:3.90.550.10_2.7.7:4.05753 |
| 2i6g | 2i6gB00:1nw3A02:3.40.50.150_2.1.1:102.717 |
| 2if2 | 2if2A00:1uf9A00:3.40.50.300_2.7.1:4.546 |
| 2igt | 2igtA01:1nw3A02:3.40.50.150_2.1.1:98.9873 |
| 2ijz | 2ijzA01:2gljA01:3.40.630.10_3.4.11:5.5089 |
| 2in3 | 2in3A01:2i3yA01:3.40.30.10_1.11.1:4.40151 |
| 2iqt | 2iqtA00:1zaiA00:3.20.20.70_4.1.2:9.86981 |
| 2nuj | 2nujA01:1s5uE00:3.10.129.10_3.1.2:63.5832 |
| 2o2g | 2o2gA00:1cex000:3.40.50.1820_3.1.1:11.9336 |
| 2o3j | 2o3jB01:1mfzA01:3.40.50.720_1.1.1:5.03281 |
| 2o5r | 2o5rA01:1j09A01:3.40.50.620_6.1.1:42.503 |
| 2oaf | 2oafA00:1s5uE00:3.10.129.10_3.1.2:63.3521 |
| 2oce | 2oceA05:1y14D02:2.40.50.140_2.7.7:7.65888 |
| 2ocz | 2oczA00:1gqnA00:3.20.20.70_4.2.1:5.67953 |
| 2oiw | 2oiwA00:1s5uE00:3.10.129.10_3.1.2:80.8835 |
| 2ojl | 2ojlA00:1nm3A01:3.40.30.10_1.11.1:6.16215 |
| 2oka | 2okaA00:1n8jA00:3.40.30.10_1.11.1:7.43465 |
| 2oo3 | 2oo3A00:1nw3A02:3.40.50.150_2.1.1:84.6677 |
| 2oq0 | 2oq0B02:1eovA01:2.40.50.140_6.1.1:6.0483 |
| 2ord | 2ordA02:2oatA02:3.40.640.10_2.6.1:5.48177 |
| 2ov9 | 2ov9D01:1s5uE00:3.10.129.10_3.1.2:25.3432 |
| 2ozv | 2ozvA01:1nw3A02:3.40.50.150_2.1.1:78.8201 |
| 2p0g | 2p0gB00:1n8jA00:3.40.30.10_1.11.1:6.61628 |
| 2p1j | 2p1jA01:1j54A00:3.30.420.10_2.7.7:33.8189 |
| 2p2s | 2p2sA02:1p9lA02:3.30.360.10_1.3.1:5.42437 |
| 2p35 | 2p35A01:1nw3A02:3.40.50.150_2.1.1:67.9253 |
| 2p7i | 2p7iA00:1xvaA02:3.40.50.150_2.1.1:59.5088 |
| 2p8j | 2p8jA00:1nw3A02:3.40.50.150_2.1.1:79.6781 |
| 2pbl | 2pblA01:1cex000:3.40.50.1820_3.1.1:8.49662 |
| 2pfs | 2pfsA01:2csxA01:3.40.50.620_6.1.1:3.90706 |
| 2pg3 | 2pg3A00:1k92A01:3.40.50.620_6.3.4:9.59807 |
| 2pim | 2pimA00:1s5uE00:3.10.129.10_3.1.2:31.266 |
| 2pkw | 2pkwA02:1nw3A02:3.40.50.150_2.1.1:99.8484 |
| 2pok | 2pokA01:1cg2A01:3.40.630.10_3.4.17:5.97712 |
| 2prx | 2prxA00:1c8uA02:3.10.129.10_3.1.2:19.8074 |
| 2py6 | 2py6A03:1nw3A02:3.40.50.150_2.1.1:79.059 |
| 2q0z | 2q0zX02:2bcqA02:1.10.150.20_2.7.7:70.2923 |
| 2q78 | 2q78F00:1s5uE00:3.10.129.10_3.1.2:31.8377 |
| 2qe6 | 2qe6A00:1yub001:3.40.50.150_2.1.1:48.8705 |
| 2qgq | 2qgqA02:1sro000:2.40.50.140_2.7.7:4.06314 |
| 2qgz | 2qgzA01:1tueD00:3.40.50.300_3.6.1:3.64559 |
| 2qh5 | 2qh5B00:1vpaA00:3.90.550.10_2.7.7:9.48071 |
| 2qjw | 2qjwA00:1cex000:3.40.50.1820_3.1.1:12.9635 |

**Supplementary Table 1 Table of FLORA predictions for PSI structures (only top hit shown, but full results in supplementary Excel table)**
